# Introductory Lectures

**Published:** 1849-02

**Authors:** 


*Introductory Lectures*—Introductory lectures have been received from James Blake, M. D., Prof. of General and Special Anatomy in St. Louis University, Missouri; Frederick Merrick, M. D., Prof. of Chemistry and Botany in Starling Medical College; Joseph Mather Smith, M. D., Prof. of Theory and Practice of Medicine, College of Physicians and Surgeons in the City of New York; James Bryan, M. D., Prof. of Surgery, Geneva Medical College. We beg to return our thanks for being remembered.

